# Visual Prognosis Following Cataract Surgery in Highly Myopic Patients with Prior History of Verisyse Phakic Intraocular Lens Implantation

**DOI:** 10.3390/jcm13164760

**Published:** 2024-08-13

**Authors:** Bosten A. Loveless, Kayvon A. Moin, Majid Moshirfar, Tyler V. Olson, Phillip C. Hoopes

**Affiliations:** 1 Hoopes Vision Research Center, Hoopes Vision, Draper, UT 84020, USA; bosten.loveless@rvu.edu (B.A.L.); kmoin@hoopesvision.com (K.A.M.); tolson@hoopesvision.com (T.V.O.); pch@hoopesvision.com (P.C.H.); 2 College of Osteopathic Medicine, Rocky Vista University, Ivins, UT 84738, USA; 3 John A. Moran Eye Center, School of Medicine, University of Utah, Salt Lake City, UT 84132, USA; 4 Utah Lions Eye Bank, Murray, UT 84107, USA

**Keywords:** Artisan Verisyse, iris-fixated, myopia, intraocular lens, visual prognosis, age-related nuclear sclerosis, ICL

## Abstract

**Background/Objectives**: This study aimed to evaluate the visual outcomes and prognosis after cataract surgery in patients with prior history of Verisyse phakic intraocular lens (pIOL) implantation. **Methods**: A retrospective cohort study involving 215 Verisyse pIOL implantations and 17 explantations was conducted. The Verisyse pIOL was disenclaved and removed through a superior scleral tunnel incision. Cataract extraction with phacoemulsification was then performed through a temporal clear corneal incision. **Results**: An occurrence rate of 7.9% of eyes with cataract formation was found. Both uncorrected (UDVA) and corrected visual acuity (CDVA) three months after cataract surgery were significantly improved (0.24 ± 0.30 vs. 0.73 ± 0.48; *p* < 0.001 and 0.10 ± 0.14 vs. 0.30 ± 0.31; *p* = 0.004, respectively). The UDVA was 20/20 or better in 41% of eyes and 20/40 or better in 65% of eyes. The CDVA was 20/20 or better in 53% of eyes and 20/40 or better in 88% of eyes. The safety and efficacy indices were 1.96 ± 1.68 and 1.60 ± 1.36, respectively. **Conclusions**: Various complications including cataracts may develop in these patients. Verisyse pIOLs have a lower incidence of cataract formation and are more likely to lead to age-related cataracts rather than the anterior subcapsular cataracts commonly seen in implantable collamer lens (ICL) patients. Patients with a prior history of Verisyse pIOL can expect to have a good visual prognosis after cataract extraction.

## 1. Introduction

For the correction of high myopia, studies have shown that phakic intraocular lenses (pIOLs) are more predictable than corneal refractive surgeries (CRSs) [1]. In addition to offering correction for a wide range of myopia (−6.0 to −20.0 D), pIOL implantation is a removable/reversible procedure and avoids the risks of corneal ectasia [2]. The Artisan Verisyse lens, an anterior chamber iris-fixated pIOL, was manufactured by Abbot Medical Optics (Santa Ana, CA, USA) and received Food and Drug Administration (FDA) approval in 2004 [3]. Studies have shown that the Verisyse pIOL provides safe and efficacious visual outcomes, predictability, and stability with few complications [4,5,6,7]. However, several complications have been reported, including corneal decompensation [8,9,10], elevated intraocular pressure [4,5,6,7], endophthalmitis [4], and retinal detachment [5,6,7]. Cataract formation has also been reported after Verisyse pIOL implantation, although the relationship between the two events is unclear. The mechanisms underlying cataract formation with posterior chamber pIOLs is documented and well understood with several studies having attempted to identify any risk factors that may explain the occurrence of cataracts after Versisyse pIOL implantation [3,11]. Cataract formation after posterior chamber pIOL implantation is thought to be due to a vault < 250 µm, resulting in a close proximity to the crystalline lens and poor aqueous humor circulation [11]. Comparatively, Verisyse pIOLs are implanted in the anterior chamber and are nowhere near the crystalline lens [3,11]. Nonetheless, cataract formation is one of the most common indications for pIOL explantation and must be considered a potential complication [3,6].

Acknowledging the uncertain relationship between cataract formation and Verisyse pIOL implantation, our objective for this study is to report the occurrence of cataract formation after Verisyse pIOL implantation and evaluate the visual outcomes and prognosis after concurrent pIOL explantation and cataract extraction (pIOL-CE).

## 2. Materials and Methods

### 2.1. Study Design

This study is a retrospective analysis of protected medical records of de-identified patients who underwent Verisyse pIOL implantation from 1999 to 2016. The consent and study procedure were approved by the Hoopes Vision Ethics Committee and adhered to the tenets of the Declaration of Helsinki. The Biomedical Research Alliance of New York (BRANY) Institutional Review Board (IRB) approved this retrospective study (#A20-12-547-823).

Inclusion criteria consisted of patients with a history of Verisyse pIOL who developed cataracts and subsequently underwent pIOL-CE and have accessible preoperative and postoperative data. A comprehensive slit lamp examination was performed to identify these patients with visually significant cataracts. Patients with a spherical equivalent (SEQ) of −15 D or greater (two patients, two eyes) had a 5 mm pIOL implanted, whereas those with a SEQ less than −15 D had a 6 mm pIOL implanted.

Preoperative biometry measurements were obtained from Lenstar LS 900 version i9.6.3.0 (Haag-Streit, Koeniz, Switzerland). The intraocular lens (IOL) model, power, and target refraction were calculated preoperatively. The SRK-T formula [12] was used for patients who underwent surgery prior to 2013 (five eyes) and both the SRK-T and the Barrett RX formulas (ASCRS, Fairfax, VA, USA) were used for patients who underwent surgery after 2013 (twelve eyes) (Table 1).

### 2.2. Surgical Techniques

Prior to surgery, all patients received a 5 cc retrobulbar injection of 4% lidocaine/0.75% bupivacaine (50:50 mixture) with 100 units of hyaluronidase to achieve akinesia and anesthesia. A single surgeon (MM) began with a 7 mm superior conjunctival peritomy near the corneal limbus. A self-sealing 5–6 mm frown scleral tunnel incision was then made in a triplanar fashion 1 mm behind the limbus at the 12 o’ clock position. The anterior chamber was then entered using a keratome. Dispersive viscoelastic was then injected into the anterior chamber to stabilize it and to protect the corneal endothelium during the disenclavation of the Verisyse pIOL. The pIOL was then rotated and removed carefully through the superior scleral tunnel incision. The surgeon and the microscope were repositioned temporally and a 2.4 mm temporal clear corneal incision as well as a continuous curvilinear capsulorhexis (CCC) were made. Using an irrigation cannula, hydrodissection and hydrodelineation were performed. The cataract and cortical lens material were subsequently removed using phacoemulsification and irrigation/aspiration handpieces. A cohesive viscoelastic agent was then injected into the capsular bag and an IOL was placed inside. Any residual viscoelastic agent was then removed. The superior scleral tunnel incisions were self-sealing in all cases. The superior conjunctival peritomy was then closed using two interrupted 10-0 Vicryl sutures (see Video S1).

Postoperative visits were conducted at one day, one week, one month, and three months after CE. Outcomes included the safety and efficacy indices. Other visual parameters measured included patients’ uncorrected distance visual acuity (UDVA), corrected distance visual acuity (CDVA), SEQ, and refractive cylinder measurements from manifest refraction.

### 2.3. Statistical Analysis

The data were analyzed using Microsoft Excel version 16.87 (Microsoft Corporation, Redmond, WA, USA). Given the small sample size of the study, lack of need for complex statistical analyses, and accessibility, Microsoft Excel was used for analysis instead of other commonly used statistics programs. The mean values, standard deviations, and one-tailed Student *t*-tests were calculated for parameters with a normal distribution. Statistical significance was set at 0.05. Normality was evaluated through a Shapiro–Wilk test. Data found to be nonparametric were evaluated and analyzed utilizing median, interquartile ranges (IQR), and the Wilcoxon/Mann–Whitney test depending on paired vs. unpaired to test for statistical significance. The nine standardized refractive surgery graphs were constructed utilizing mEYEstro software version 2.3 (MathWorks Inc., Natick, MA, USA) [13].

## 3. Results

### 3.1. Demographics

A total of 122 patients (215 eyes) underwent Verisyse pIOL implantation from 1999 to 2016. Within this cohort, 13 patients (17 eyes, 9 females, 4 males) developed visually significant cataracts between 2012 and February 2024 requiring removal, resulting in an estimated occurrence of 7.9%. The average age of our explanted group at the time of pIOL-CE was 61.15 ± 7.23 years (range: 46 to 71). The average age of the entire population at this point in time was 57.7 years (range: 41 to 77) (Table 1).

**Figure 1:**
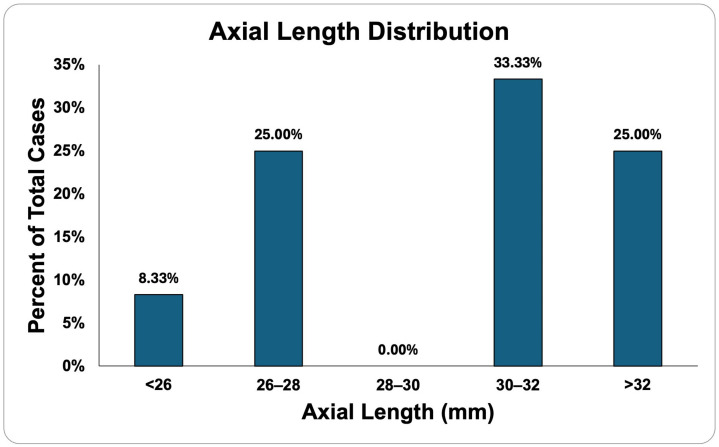
Axial length distribution of explanted eyes.

All eyes (17/17) had nuclear sclerotic (NS)-type cataracts. Additional posterior subcapsular (PSC) components were observed in 41% of eyes, while anterior subcapsular (ASC) and cortical spoke (CS) components were each found in 6% of eyes. Concerning NS, 94% of cases were grade 2+ or more. The preoperative biometric findings of the patients who underwent pIOL-CE are included in Table 1. The mean axial length was 29.58 mm ± 2.93 (range: 26.07 to 33.7). See Figure 1 for further information on the distribution of axial lengths.

One patient (one eye) developed a retinal detachment due to pathologic myopia and received a vitrectomy eight months prior to pIOL-CE. The patient’s other eye has not developed a cataract at this point in time. Another patient (one eye) had corneal decompensation and therefore had a concurrent descemet’s stripping automated endothelial keratoplasty (DSAEK) during pIOL-CE.

All IOL models placed were monofocal. Three-piece IOLs were used in 42% (5/12) of eyes, with the rest being one-piece IOLs. Toric lenses were utilized in 17% (2/12) of eyes. There were six different IOL models in total (Table 2).

### 3.2. Visual Outcomes

**Figure 2:**
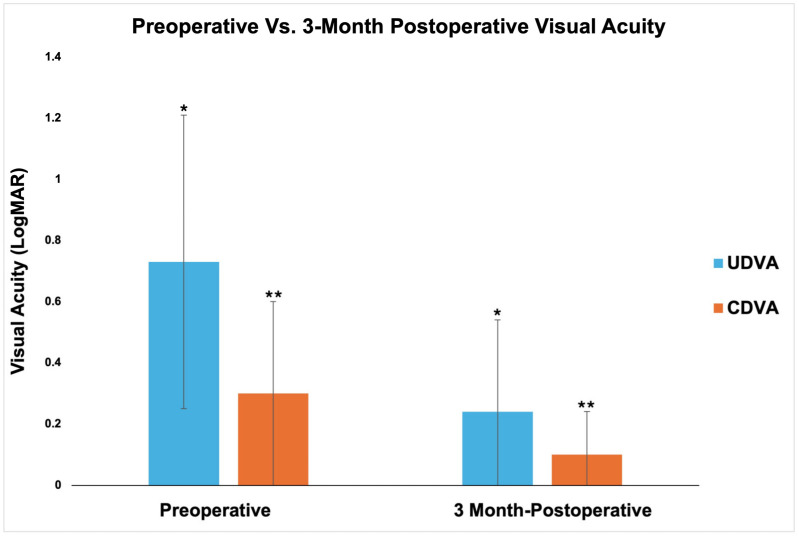
Preoperative vs. 3-month postoperative visual acuity. */** = statistical significance *p* < 0.05 between respective groups. Abbreviations: UDVA, uncorrected distance visual acuity; CDVA, corrected visual acuity.

Multiple comparisons between preoperative and postoperative visual acuity at one day, one week, and three months demonstrated significant reduction in logMAR values (*p* < 0.015). One-day and one-week compared to three-month postoperative time points (*p* < 0.030) as well as UDVA one-month to three-month postoperative time points (*p* = 0.035) showed significant improvement in visual acuity (Table 3 and Figure 2).

**Figure 3:**
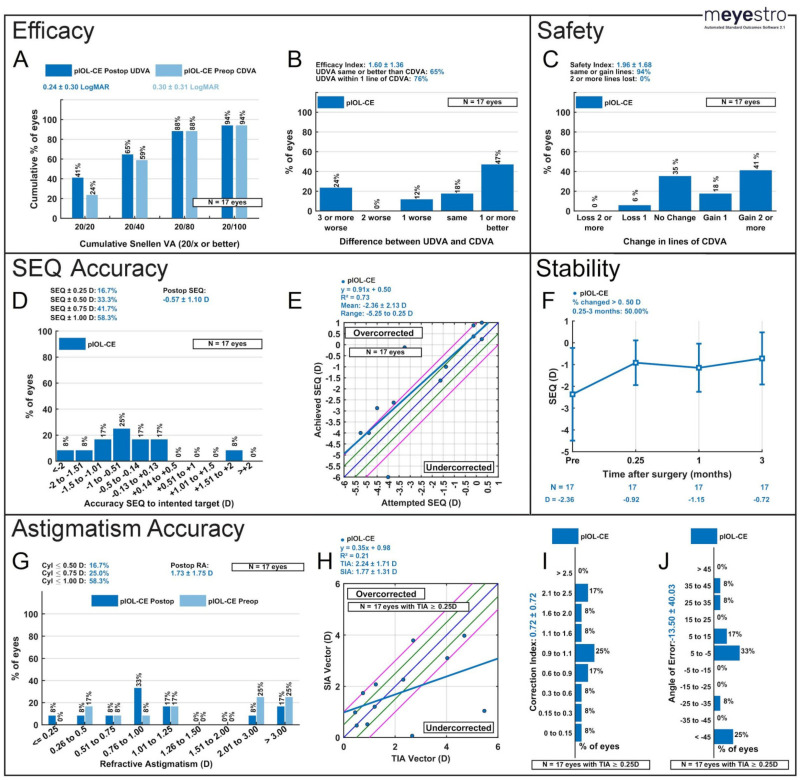
Nine standardized refractive surgery graphs at three months after CE. (**A**) Postoperative uncorrected distance visual acuity (UDVA) versus preoperative corrected distance visual acuity (CDVA). (**B**) Efficacy: Change in Snellen lines from preoperative CDVA to postoperative UDVA. (**C**) Safety: Change in Snellen lines from preoperative CDVA to postoperative CDVA. (**D**) Spherical Equivalent (SEQ) Accuracy: Accuracy of postoperative spherical equivalent refraction to target. (**E**) Attempted versus achieved spherical equivalent refraction, with linear regression and correlation values; the black line represents the equation y = x, and the closer the regression line is to the black line, the more accurate the results. (**F**) SEQ stability; (**G**) cylindrical astigmatism accuracy; (**H**) TIA vs. SIA; blue line: target; between green lines: within ±0.50 D of target; between pink lines: within ±1.0 D of target. (**I**) Histogram of correlation index; (**J**) angle of error. CE = Cataract extraction; TIA = target-induced astigmatism; SIA = surgically induced astigmatism; ICL = implantable collamer lens; D = diopter.

The cumulative Snellen UDVA was 20/20 or better in 41% of eyes and 20/40 or better in 65% of eyes at three months. Postoperative UDVA at three months was the same or better than preoperative CDVA in 65% of eyes. (Figure 3A,B).

Additionally, postoperative UDVA was within one line of preoperative CDVA in 76% of eyes. Postoperative UDVA was the same or better than the preoperative CDVA in 65% of eyes at three months. CDVA was the same or better in 94% of the eyes three months postoperatively and 6% of eyes lost one line of vision (see Figure 3C).

The safety and efficacy indices three months after pIOL-CE were 1.96 ± 1.68 and 1.60 ± 1.36, respectively.

### 3.3. Refractive Outcomes

A total of 58.3% of eyes (7/17) were ± 1 D at three months postoperatively and 16.7% (2/17) eyes were within ± 0.25 D (see Figure 3D). SEQs ranged from −3.125 to +2 D with only two eyes outside of ± 1 D. The attempted vs. achieved SEQ vector had a mean of −2.36 ± 2.13 D ranging from −5.25 to 0.25 D and had an R^2^ = 0.73 (see Figure 3E). From one week to three months postoperatively, 50% of eyes had more than a 0.50 D change in SEQ (see Figure 3F).

Although astigmatism correction was not the main objective for these patients and only two eyes had toric lenses placed, we still evaluated astigmatism correction. At three months, 58.3%, 25.0%, and 16.7% of eyes had a refractive cylinder ≤ 1.0, 0.75, and 0.50, respectively. The mean preoperative cylinder was −2.40 ± 1.88 D, whereas the postoperative mean was −1.73 ± 1.75 D (Table 3, Figure 3G). The target-induced astigmatism (TIA) vector is reported in Figure 3H as being 2.24 ± 1.71 D and the surgically induced astigmatism (SIA) vector is 1.77 ± 1.31 D with an R^2^ = 0.21. Additional findings included a correction index of 0.72 ± 0.72, as seen in Figure 3I, and an angle of error of −13.50 ± 40.03°, with 50% of eyes falling between −25 and 25° (Figure 3J).

**Figure 4:**
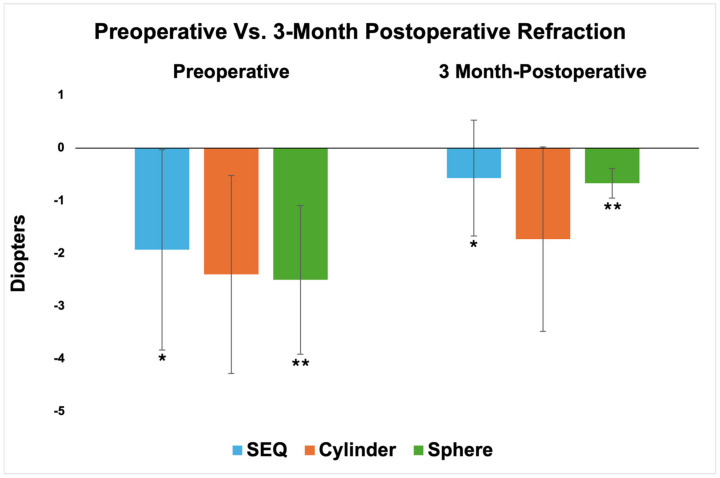
Preoperative vs. 3-month postoperative refractive values. */** = statistical significance *p* < 0.05 between respective groups. Abbreviations: SEQ, spherical equivalent.

The mean SEQ was −0.57 ± 1.10 D and the mean sphere was −0.67 ± 0.28 D at three months. These values are significantly closer to plano than preoperatively (*p* = 0.007 and *p* = 0.006, respectively) (Table 3 and Figure 4). There was no significant change in cylinder.

## 4. Discussion

Although uncommon, visually significant cataract formation has been reported after Verisyse pIOL implantation [7]. Our study demonstrated that the occurrence of cataract formation in our cohort of patients was approximately 7.9%, whereas other studies have reported occurrences ranging from 1.5 to 11.1% [7,14]. In comparison, ICLs have a higher rate of cataract formation than Verisyse pIOLs (18.3% in 10 years), with the most common type of cataract seen being ASC (83.3%) [15]. Despite these documented occurrences, the relationship between cataract formation and Verisyse pIOLs is unclear. Some authors propose that inflammation after uneventful implantation, crystalline lens trauma during surgery, or insufficient aqueous humor circulation (ICL hole flow is 1.7 to 39.6%, Verisyse iridotomy flow is 79 to 95.5%) can be potential factors for cataract formation in these patients [14,16,17]. However, there were no intraoperative complications noted for our patients to suggest any potential trauma or any postoperative signs of inflammation. Computer simulation studies using iris-fixated pIOLs have additionally shown that altered aqueous humor circulation is an unlikely cause for cataract formation [17]. We believe that the cataract formation in our cohort was likely age-related than pIOL-related due to the presence of several confounding risk factors described in the following paragraph.

The patients in our cohort that developed cataracts were on average 4.31 years older than the rest of the cohort. Additionally, the mean age at the time of pIOL-CE was 61.15 ± 7.23 years. In comparison, the mean age of patients undergoing ICL explantation due to cataract formation was 41 ± 7 years in one study [18]. Given that the reported 10-year incidence of cataract surgery in adults aged 55–64 is 9.1% [19], and considering that the average age at the time of surgery is approximately 65 [20], it is very likely that cataract formation in our patient sample was age-related. Another risk factor arguing for an age-related etiology is that the average AL of our patient sample population was 29.58 ± 2.93 mm (range: 26.07 to 33.7), revealing that these patients were severely myopic prior to pIOL implantation. High myopia is a well-known risk factor for age-related nuclear cataract formation. One study found that patients with longer axial lengths (>24.00 mm) and high myopia (≥6.00 D) had an earlier incidence and higher grade of NS cataracts [21]. In alignment with these findings, all of our patients (100%) were found to have NS cataracts.

Overall, our visual outcomes were very similar to those reported by other studies. While some studies excluded patients with vision-threatening diseases (i.e., keratoconus, retinal detachment, central serous chorioretinopathy, macular edema, glaucoma, and choroidal neovascularization), we opted to include these patients in our study. Omoto et al. analyzed a sample of 96 eyes, with 53 of them implanted with PMMA phakic IOLs, similar to our patient population. Their UDVA improved from 0.29 ± 0.34 to 0.03 ± 0.19 logMAR, CDVA went from −0.01 ± 0.17 to −0.09 ± 0.10 logMAR, and SEQ improved from −1.43 ± 1.59 to −0.17 ± 0.84 D at one month postoperatively. Safety and efficacy values were 1.02 ± 0.56 and 1.31 ± 0.64, respectively [22]. Although baseline comparisons are more difficult due to differing exclusion criteria, our study had slightly better visual outcomes. This is interesting since we included vision-threatening diseases in our analysis. Another study by de Vries et al., which had a smaller sample size (n = 36 eyes) and included participants with vision-threatening diseases, focused solely on evaluating refractive and visual outcomes. Their CDVA improved from 0.45 ± 0.38 to 0.17 ± 0.18 logMAR and had an SEQ of −0.28 ± 1.11 at 6 months postoperatively [23]. Of notable mention was a more generalized study by Leccisotti et al. that included two eyes implanted with iris-fixated lenses. One eye had an ASC cataract with a final postoperative SEQ of −0.50 D. The other eye had an NS cataract with a final postoperative SEQ of −0.75 D [24].

Unquestionably, iris-fixated lens explantations are challenging. We believe that our technique allows for the simultaneous removal of the lens during cataract surgery without inducing significant astigmatism or complications such as leakage from the scleral incision. By separating the two incisions, this surgery becomes more similar to a simple cataract surgery, allowing less experienced surgeons to still perform it on these patients. Given the frequency of surgeries that this population undergoes, as well as the propensity for iris-fixated lenses to cause endothelial cell loss, an overall lower-baseline endothelial-cell density at the time of cataract surgery must be taken into consideration. Cell loss is more likely to occur in iris-fixated pIOLs than with ICLs (Verisyse 10.3%, ICL 3.5% cell loss after three months) [25]. Other studies using iris-fixated lenses have found that a 1.83% average cell loss occurs in the first year post implantation, with a 0.87% subsequent average loss each year [26]. Additionally, it has been shown that around three months after phacoemulsification alone that an 11.8% loss of endothelial cells occurred [27]. Continual follow-up care and monitoring for potential corneal decompensation after cataract surgery in this population are necessary. Although evaluating corneal decompensation in this patient subset was not our main objective, our preoperative specular microscopy in 47% (8/17) of eyes showed an average baseline of 1955 ± 554 cells prior to pIOL-CE. Fortunately, only one of our patients had a significant enough cell loss preoperatively to merit concurrent DSAEK.

All retrospective studies have some limitations due to inherent selection bias. Our study was further limited by a small sample size and confounding variables in visual acuity such as retinal detachment. We were also forced to limit our postoperative time points due to some patients that were lost to follow-up. Our cataract occurrence rate may also have been affected by loss of follow-up after pIOL implantation.

Because of the nature of this surgery, astigmatism was not corrected in most patients. Only (2/17) eyes of our study group had toric IOLs placed and the mean residual cylinder was reduced overall by 0.65 D. The large scleral incision made during this surgery prevented any induced astigmatism that could occur from a corneal incision. Accordingly, our results show that there was no significant change in the refractive cylinder.

## 5. Conclusions

Verisyse pIOLs are an effective and predictable option for patients with high levels of myopia. Although Verisyse pIOLs have a relatively low complication rate, risk still exists. Continual follow-up care and monitoring is necessary, especially for corneal decompensation since these patients are at higher risk. Compared to ICLs, Verisyse pIOLs have a lower occurrence rate of cataract formation. Patients can be counseled that there is a lower risk of cataracts from Verisyse pIOL placement or long-term use. If removal becomes necessary, clinicians can be aware that the impact on visual acuity and SEQ will be minimal.

## Figures and Tables

**Table 1:** Preoperative characteristics and biometric findings prior to pIOL-CE.

Variable	Mean (±SD)	Range
Sex	65% Female	35% Male
Age at time of implant (Years)	45 ± 8	33 to 53
Age at time of pIOL-CE (Years)	61 ± 7	46 to 71
Time between surgeries (Years)	15.75 ± 3.90	4 to 18
Sphere (D)	−2.50 ± 1.41	−5.00 to −1.00
Cylinder (D)	−2.40 ± 1.88	−6.00 to −0.50
AL (mm)	29.58 ± 2.93	25.68 to 33.70
Km (D)	44.59 ± 3.19	41.07 to 52.06
WTW (mm)	12.40 ± 0.37	11.94 to 13.08
ACD (mm)	3.21 ± 0.66	2.17 to 4.25
AQD (mm)	2.78 ± 0.82	1.56 to 4.16
CCT (μm)	544 ± 57	440 to 642
IOL Sphere (D)	5.00 ± 3.50	1.00 to 11.50
ECD (cell count)	1955 ± 554 *	888 to 2484 *

Abbreviations: SD = standard deviation; pIOL-CE = phakic intraocular lens implantation-cataract extraction; AL = axial length; Km = mean keratometry; WTW = white-to-white; ACD = anterior chamber depth; AQD = aqueous depth; CCT = central corneal thickness; IOL = intraocular lens; ECD = endothelial cell density; D = diopters; * n = 8.

**Table 2:** Lens models and types.

Company	Model	Lens Type
Bausch + Lomb (Laval, Canada)	MX60E (3)	hydrophobic acrylic 1-piece
Johnson & Johnson (New Brunswick, NJ, USA)	AR40E (4), AR40M (1)	hydrophobic acrylic 3-piece
	ZCU375 (1), ZCT400 (1)	hydrophobic acrylic 1-piece toric
	ZCB00 (2)	hydrophobic acrylic 1-piece

**Table 3:** Preoperative vs. 3-month postoperative visual and refractive outcomes.

Variable	Preoperative M ± SD (R)	3-Month Postoperative M ± SD (R)	*p*
UDVA (LogMAR)	0.73 ± 0.48 (0.18 to 1.85)	0.24 ± 0.30 (0.00 to 1.00)	**<0.001**
CDVA (LogMAR)	0.30 ± 0.31 (0.00 to 1.18)	0.10 ± 0.14 (0.39 to 0.10)	**0.004**
SEQ (D)	−1.93 ± 1.90 (−5.25 to +0.25)	−0.57 ± 1.10 (−3.125 to +2.00)	**0.007**
Cylinder (D)	−2.40 ± 1.88 (−6.00 to −0.50)	−1.73 ± 1.75 (−6.00 to 0.00)	0.087
Sphere (D)	−2.50 ± 1.41 (−5.00 to −1.00)	−0.67 ± 0.28 (−1.00 to −0.25)	**0.006**

Bolded text indicated statistical significance. Abbreviations: UDVA, uncorrected distance visual acuity; CDVA, corrected distance visual acuity; SEQ, spherical equivalent; D, diopters; M, mean; SD, standard deviation; R, range.

## Data Availability

The data presented in this study are available on request from the corresponding author due to ethical restrictions.

## Supplementary Materials

The following supporting information can be downloaded at: https://www.mdpi.com/article/10.3390/jcm13164760/s1, Video S1: Simultaneous Verisyse Phakic IOL explantation with cataract extraction using phacoemulsification and posterior chamber IOL implantation.
